# Chytrid fungus infections in laboratory and introduced *Xenopus laevis* populations: assessing the risks for U.K. native amphibians

**DOI:** 10.1016/j.biocon.2015.01.034

**Published:** 2015-04

**Authors:** Richard C. Tinsley, Peter G. Coxhead, Lucy C. Stott, Matthew C. Tinsley, Maya Z. Piccinni, Matthew J. Guille

**Affiliations:** a School of Biological Sciences, University of Bristol, Bristol BS8 1TQ, UK; b European Xenopus Resource Centre, School of Biological Sciences, University of Portsmouth, Portsmouth PO1 2DT, UK; c School of Biological and Environmental Sciences, University of Stirling, Stirling FK9 4LA, UK

**Keywords:** African clawed frog, Chytrid fungus (*Bd*), Emerging infectious disease (EID), Global spread of pathogens, Invasive species, Threats to native species

## Abstract

The chytrid fungus *Batrachochytrium dendrobatidis* (*Bd*) is notorious amongst current conservation biology challenges, responsible for mass mortality and extinction of amphibian species. World trade in amphibians is implicated in global dissemination. Exports of South African *Xenopus laevis* have led to establishment of this invasive species on four continents. *Bd* naturally infects this host in Africa and now occurs in several introduced populations. However, no previous studies have investigated transfer of infection into co-occurring native amphibian faunas. A survey of 27 U.K. institutions maintaining *X*. *laevis* for research showed that most laboratories have low-level infection, a risk for native species if animals are released into the wild. RT-PCR assays showed *Bd* in two introduced U.K. populations of *X*. *laevis*, in Wales and Lincolnshire. Laboratory and field studies demonstrated that infection levels increase with stress, especially low temperature. In the U.K., native amphibians may be exposed to intense transmission in spring when they enter ponds to spawn alongside *X*. *laevis* that have cold-elevated *Bd* infections. Exposure to cross-infection has probably been recurrent since the introduction of *X*. *laevis*, >20 years in Lincolnshire and 50 years in Wales. These sites provide an important test for assessing the impact of *X*. *laevis* on *Bd* spread. However, RT-PCR assays on 174 native amphibians (*Bufo*, *Rana*, *Lissotriton* and *Triturus* spp.), sympatric with the *Bd*-infected introduced populations, showed no foci of self-sustaining *Bd* transmission associated with *X*. *laevis*. The abundance of these native amphibians suggested no significant negative population-level effect after the decades of co-occurrence.

## Highlights

• We addressed the potential for transmission of chytrid fungus (Bd) by *Xenopus laevis*.
• A survey of laboratory colonies of *X*. *laevis* in the U.K. found most infected with Bd.
• Bd infects *X*. *laevis* in the wild in the U.K., 50 years after original host introduction.
• Parallel surveys found no Bd in native amphibians sympatric with infected *X*. *laevis*.
• Interactions/outcomes may differ in other global regions but robust data are needed.

## 1 Introduction

Infection of amphibians by the chytridiomycete fungus *Batrachochytrium dendrobatidis* (*Bd*) is currently responsible for a major pandemic whose lethal pathological effects are implicated in widespread extinctions of susceptible host populations. Its epidemiology may create waves of infection accompanied by overwhelming death rates (Cheng et al., 2011; Lips et al., 2006). The origin of this emerging infectious disease (EID) is much debated: interpretations of sudden appearance globally include the ‘novel pathogen hypothesis’ (emphasising anthropogenic dispersal) and the ‘endemic pathogen hypothesis’ (supposing increased susceptibility to pre-existing infection triggered by environmental changes); pathogen hypervirulence may also have evolved recently via recombination/hybridisation between *Bd* lineages that were historically isolated (e.g. Farrer et al., 2011; Morgan et al., 2007). Global dissemination is considered to have been facilitated by trade in amphibians (for food, sport, the pet trade and laboratory use) (Fisher and Garner, 2007). Several amphibian species have been recognised as carriers of infection including the North American bullfrog, *Lithobates catesbeianus*, and the African clawed frog, *Xenopus laevis*, both of which have established in novel geographical regions following anthropogenic introduction (Fisher and Garner, 2007).

*Xenopus laevis* has been implicated in an ‘out-of-Africa’ origin for the *Bd* pandemic (Weldon et al., 2004) although recent evidence indicates a more complex history and global evolution (Farrer et al., 2011; Rosenblum et al., 2013). *Bd* is a widely-distributed infection of *X*. *laevis* (see Soto-Azat et al., 2010) and hosts can develop a powerful immune response, typically without significant pathology, so individuals survive to disseminate their infection to co-occurring susceptible species (Rollins-Smith et al., 2009). *Bd* is endemic in southern Africa across the native range of *X*. *laevis* and this anuran has been exported almost worldwide for use in biological and biomedical laboratories (Gurdon, 1996; Tinsley, 2010). There is historical evidence from museum collections that *Bd* was present in *X*. *laevis* in the Western Cape, South Africa in the late 1930s coinciding with the start of major exports from this region (Weldon et al., 2004): this route of potential global transfer has continued up to the present. Typically, where introduced into new regions, *X*. *laevis* has been regarded as an invasive species. Release or escape of captive animals (maintained in lab populations and/or as pets) has led to spread of *X*. *laevis* in 4 continents in the past half century. Introduced populations are recorded in the U.S.A., Chile, France, Portugal, Italy, U.K. and Japan together with isolated reports in other European countries and on Ascension Island (Measey et al., 2012; Tinsley and McCoid, 1996). While few field studies have been undertaken into the inter-specific interactions of *X*. *laevis* in invaded areas, there are claims of significant negative effects on native amphibians and fishes (e.g. Amaral and Rebelo, 2012; Lafferty and Page, 1997; Lillo et al., 2011). Introductions of disease agents include *Bd* in Japan (Goka et al., 2009), Chile (Solis et al., 2010) and California (Vredenburg et al., 2013) and several parasitic helminths in California (Lafferty and Page, 1997; Kuperman et al., 2004) and the U.K. (Jackson and Tinsley, 2001; Tinsley and Jackson, 1998). Although international movements of *X*. *laevis* are considered a major factor in the world spread of *Bd*, there are apparently no records of transfer of *Bd* into native species living alongside introduced *X*. *laevis*. These ‘alien’ populations provide important case studies of *Bd* spread because, in several instances, the date and location of original introduction are known, so potential effects can be followed against a temporal and spatial scale.

The introduced populations of *X*. *laevis* in the U.K. are amongst the longest established in the present world distribution. Thus, introductions in Wales and the Isle of Wight (the latter now probably extinct) were contemporaneous with the major invasions in California in the early 1960s (Tinsley and McCoid, 1996). If *Bd* had been present in these founding populations, long-term co-occurrence with native amphibians might have generated sustained pressure for inter-species transfer, potentially resulting in new endemic disease in native species or in their local extinction. In laboratory studies, cold temperatures immunosuppress *X*. *laevis*, increasing susceptibility to *Bd* infection (Rollins-Smith et al., 2009, 2011), so the U.K. populations might be an especially potent source of *Bd* transmission. Thus, in Wales, *X*. *laevis* occurs at sites where water temperatures are below 10 °C for over half of the year (Tinsley et al., 2011a) and habitat sharing with native amphibians will be maximal at low temperatures in spring when local species enter ponds to spawn.

These circumstances, of long-term sympatry with native amphibians at low temperatures, provide the rationale for the present study. First, we surveyed *Bd* infection in U.K. laboratories maintaining *X*. *laevis* for research to assess the risk that release of infected hosts might introduce *Bd* into the wild. Second, we carried out laboratory experiments to examine environmental effects, especially temperature, on *Bd* infection in *X*. *laevis*. Third, we carried out fieldwork across the locations of the two currently-known U.K. populations of *X*. *laevis*, in Wales and Lincolnshire, to assay *Bd* infection in *X*. *laevis* and in sympatric native amphibians. Part-way through these field studies, both populations of *X*. *laevis* went extinct following two successive winters of extreme low temperatures (2009/2010 and 2010/2011). In the following two years, fieldwork was undertaken to confirm the disappearance of *X*. *laevis* (see Tinsley et al., submitted for publication) including further tests for *Bd* in native amphibians to determine whether the now-extinct populations of *X*. *laevis* had left a legacy of infection established in native species. Our results are relevant to conservation management decisions both for evaluation of the negative impact of *X*. *laevis* as an invasive species and for the design of measures to control the spread of *Bd*.

## 2 Materials and methods

To test amphibians for *Bd*, the ventral skin surface was swabbed following the procedures of Hyatt et al. (2007), including single use of sterile gloves and storage of swabs dry (at room temperature, ca. 20 °C) until testing. Protocols for *Bd* assay by real-time PCR using TaqMan probes followed Boyle et al. (2004) and Hyatt et al. (2007). Amplification standards of 1000, 100, 10, 1 and 0.1 genome equivalents (a kind gift of Mat Fisher, Imperial College London) were included in the TaqMan-based assays. PCRs, which were performed using a 1/10 dilution of DNA samples extracted from the swabs, were replicated twice initially then twice more if only one replicate amplified. A subset of PCR products was sequenced to ensure that the correct DNA had been amplified. Samples above 0.1 genome equivalents (GE) were considered positive and estimates of GE are cited in the Results (Section 3) as a measure of infection intensity.

Swabs from *X*. *laevis* maintained in U.K. research institutions were tested for infection by RT-PCR assay (*n* = 1795 swabs/27 laboratory populations). Studies on the behaviour of *Bd* under laboratory conditions included temperature effects on infection. These experiments employed *X*. *laevis* bred from parents imported from the Western Cape (environs of Cape Town), South Africa: offspring from a single spawning (designated ‘Sib 17’ in Tinsley et al., 2011b) were raised under the same conditions (at 20 °C) until metamorphosis and then maintained at 10 or 20 °C for > 6 months before testing for *Bd*. Samples of *X*. *laevis* wild-caught at Croescwtta pond, Wales, were transferred to laboratory conditions (at 20 °C) and subsequent change in *Bd* infection assessed. Procedures involving *X*. *laevis* were carried out under U.K. Home Office licence.

Fieldwork was based in a wooded valley, the Afon Alun, and adjacent farmland in Mid-Glamorgan, Wales, and on industrial wasteland, the site of a former steelworks, north of Scunthorpe, Lincolnshire. Collection of *X*. *laevis* employed baited traps set in water (see Supplementary information). In Wales, all animals contributed to a long-term mark-recapture programme continuing from 1981 to 2010 (e.g. Tinsley et al., 2012). Samples of the recaptured individuals were swabbed for assay of *Bd* before release at the capture locality. In Lincolnshire, fieldwork was associated with an eradication programme instituted by Natural England in 2003, so samples provided ‘one-off’ records of *Bd* infection in individuals that were then removed from the population.

Age determination for *X*. *laevis* in both locations was based on counts of annual growth rings in bone (see Supplementary information). In the Welsh population, age was also determined from known birth-year of individually-marked animals. Water temperatures were recorded at depths of 30–60 cm with Tinytag data loggers (Gemini (U.K.) Ltd) (Tinsley et al., 2011a) during 2007–2008 in Wales and 2004–2008 in Lincolnshire.

Most tests for *Bd* in native amphibians involved animals caught in traps set in water for *X*. *laevis*. Some native anurans were swabbed after capture on land, close to trapping sites; one sample in Wales was found away from water in a disused quarry. Common and palmate newts (*Lissotriton vulgaris* and *Triturus helveticus*) were not distinguished; great crested newts (*T*. *cristatus*) were found only at the Welsh locality (2 individuals at Croescwtta): so, data refer to combined totals of newts. Numbers of native amphibians recorded during fieldwork did not form part of a quantitative survey; data cited below provide ‘snapshots’ of occurrence as illustrations of the frequency of encounters during studies focused primarily on *X*. *laevis*.

Statistical analyses were conducted in R version 3.0.2 (R Development Core Team, 2013) using the glm function. Field data included infection records for *X*. *laevis* from a single population in Wales sampled on two occasions. The effect of sample date, host sex and host age on the likelihood of *Bd* infection was investigated using a binomial model. The model initially included these three main effects, as well as sample-date × sex and sample-date × age interactions, to test for inconsistency in the effects of sex and age across the two sample dates. For the infected cohort, the same model structure was used to investigate the effects of these terms on mean infection intensity, although in this case the response variable was the natural log of the intensity estimate and the model employed a quasipoisson error distribution. Some animals were captured on both sample dates (*n* = 17 and *n* = 5 for prevalence and intensity data-sets respectively); to avoid pseudoreplication, either the first or second capture of these individuals was randomly excluded from analyses. Statistical results presented were taken from models that had been sequentially simplified to remove non-significant terms. Additionally, a Chi-squared contingency test employing Yates’ correction was used to compare prevalence of infection between animals reared at 10 and 20 °C in the laboratory.

## 3 Results

### 3.1 Laboratory studies

**Fig. 1:**
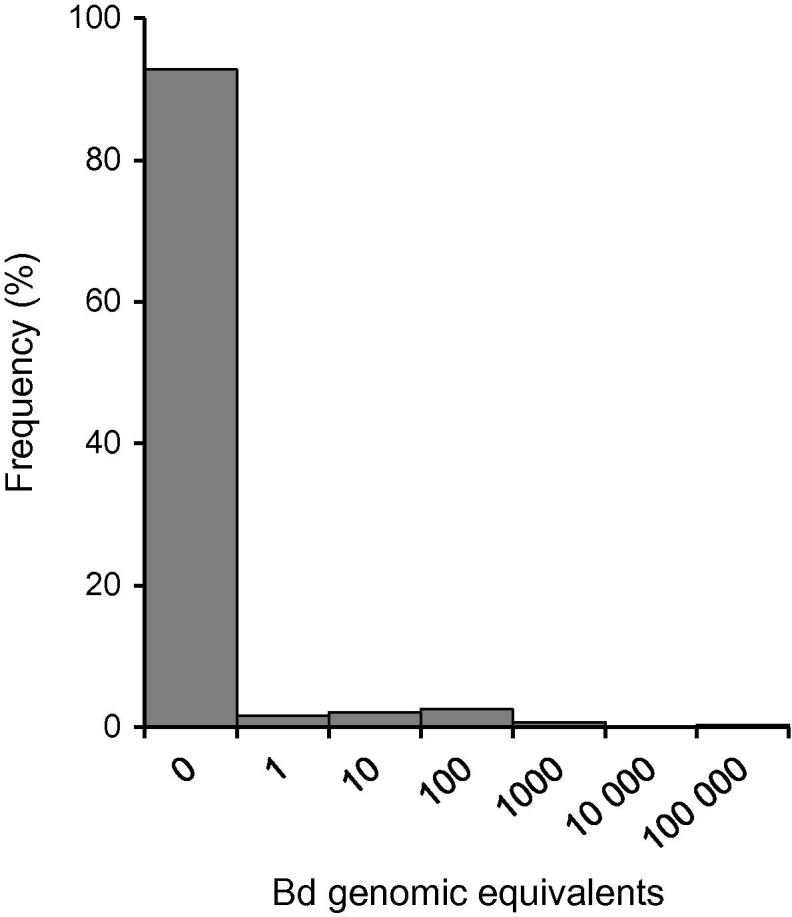
Frequency distribution of *Batrachochytrium dendrobatidis* (*Bd*) infection intensities (genomic equivalents determined by RT-PCR) in *Xenopus laevis* from U.K. laboratory populations (*n* = 1795 individuals). *X*-axis scale gives maximum value for histogram bin ranges.

To survey *Bd* infections in research institutions, samples were assayed from 27 U.K. laboratories. Twenty-four of these colonies (88.9%) carried infection: 22 with low levels (⩽100 genomic equivalents, GE) in a small subset of animals. Overall, in the combined data set, 1666 individuals were negative and 129 (7.2%) positive for *Bd*; intensity of infection was ⩽100 GE in 88% of infected individuals. Two colonies had substantial infection levels and both had recently experienced identifiable stress: a major temperature fluctuation in one and a nematode infection in the other. Amongst these, 3 individuals had GE estimates >10,000 (Fig. 1). There was no pattern identifiable relating to maintenance procedures in different laboratories, whether animals were kept in ‘flow-through’ or ‘fill-and-dump’ systems (see Tinsley, 2010) (Supplementary Table 1). Repeat testing of lab-maintained populations indicated that infection was undetectable in most animals even where these shared aquaria with others that were *Bd* positive. Supplementary Table 2 shows data for 32 individuals, each tested 11 times during 19 weeks. On 4 of the weeks when tested, the entire population was *Bd* negative. Infection was detected in 9 tests (out of the total of 352 tests); on 6 of the weeks when infection was detected, only a single individual was *Bd* positive (a prevalence of 3%) and, generally, this individual was *Bd* negative at the next test. In 2 of the 5 individuals that tested positive during the trial, their infection recurred (9–10 weeks after the previous infection), and in 1 case this relapse was prolonged (over the final 7 weeks of monitoring) (Supplementary Table 2).

**Fig. 2:**
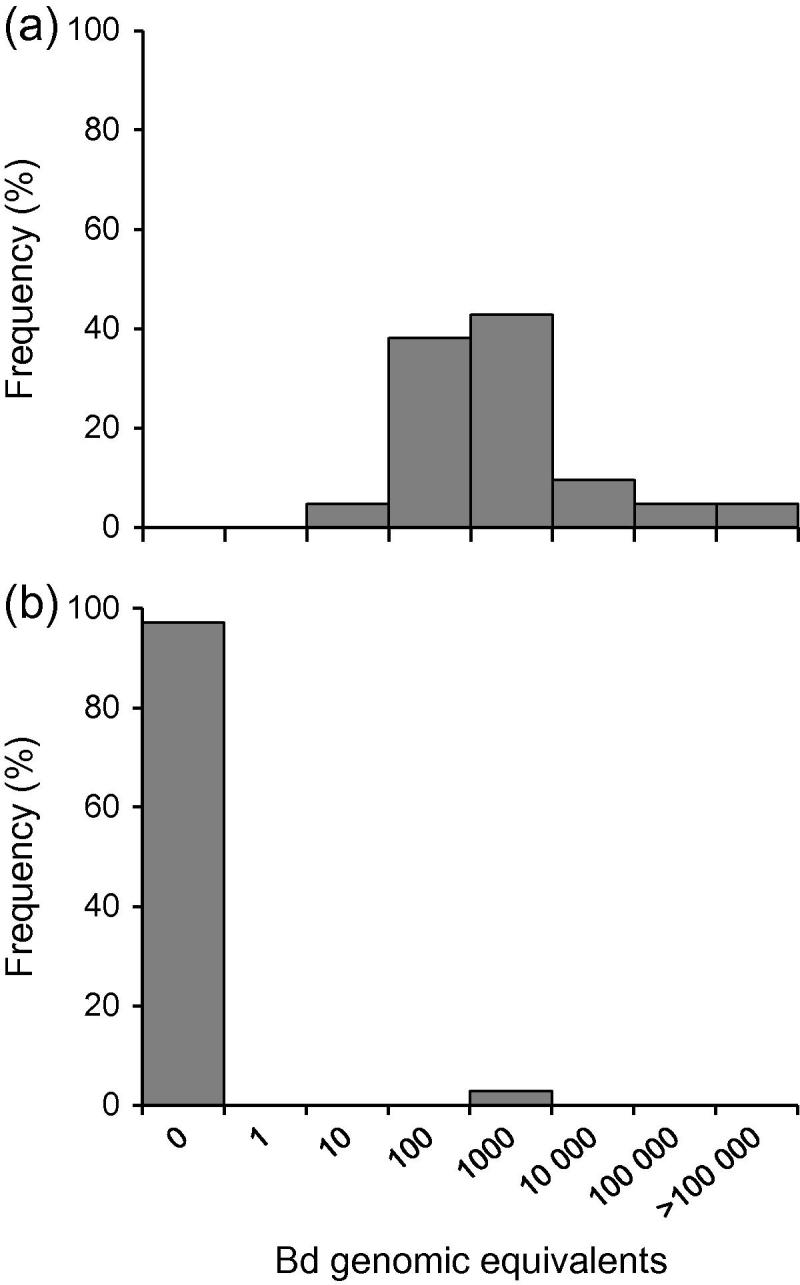
Frequency distributions of *Batrachochytrium dendrobatidis* (*Bd*) infection intensities (genomic equivalents determined by RT-PCR) in laboratory-raised *Xenopus laevis* maintained (a) at 10 °C (*n* = 22), and (b) at 20 °C (*n* = 35). *X*-axis scale gives maximum value for histogram bin ranges.

**Fig. 3:**
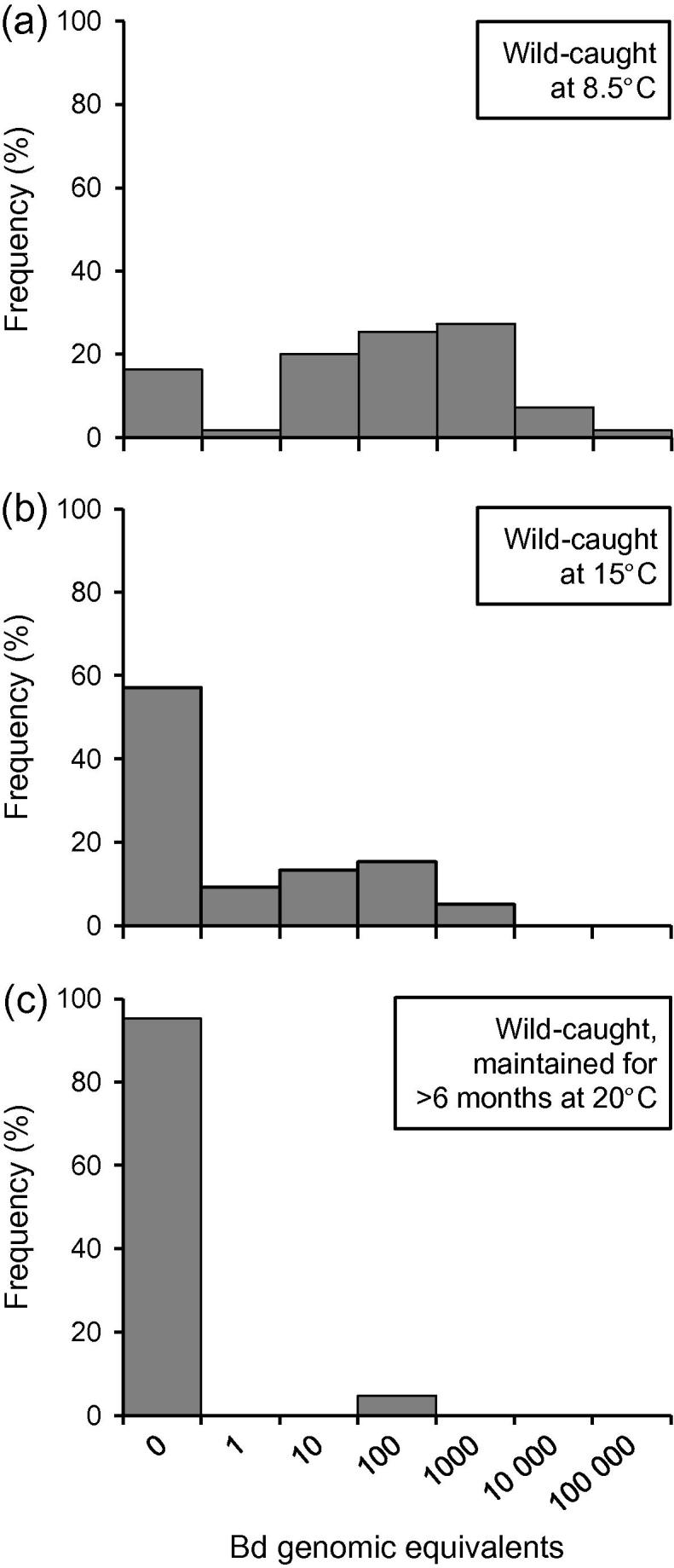
Frequency distributions of *Batrachochytrium dendrobatidis* (*Bd*) infection intensities (genomic equivalents determined by RT-PCR) in samples of *Xenopus laevis* from the same field population (Croescwtta) in Wales (a) at 8.5 °C in April (*n* = 55) and (b) at 15 °C in July (*n* = 97), and (c) after transfer to laboratory maintenance at 20 °C for >6 months (*n* = 21). *X*-axis scale gives maximum value for histogram bin ranges.

In tests of effects of temperature on *Bd* infection, lab-raised *X*. *laevis* maintained at 10 °C (*n* = 22) had 100% prevalence of *Bd* with maximum intensity >100,000 GE (Fig. 2a). Those maintained at 20 °C (*n* = 35) comprised 34 negative for *Bd* and 1 with a GE estimate of 386 (Fig. 2b). The prevalence differed significantly between these temperature regimes (*X*^2^_(1)_ = 35.43; *P* < 0.0001); infection intensity differed very markedly, but was not compared statistically because only a single individual tested positive at 20 °C, providing no replication. In the group of wild-caught *X*. *laevis* (*n* = 21), transferred to the laboratory from a Welsh population known to carry *Bd* (Section 3.2.1.), 20 were negative and 1 was positive (25 GE) after >6 months at 20 °C (Fig. 3c).

### 3.2 Field studies in Wales

#### 3.2.1 Occurrence of infection in *Xenopus laevis*

Skin swabs were tested by RT-PCR in 2 samples of *X*. *laevis* from the population at Croescwtta pond, Wales (Fig. 3a and b), caught in April (*n* = 55) and July (*n* = 97) 2008, together with the last survivor found at this site in May 2010. Prevalence of infection was 83.6% in April and 43.3% in July; the last survivor was Bd negative. Animals were selected at random for *Bd* testing, so representation of the same marked individuals in successive samples occurred by chance. Seventeen individuals were tested in both April and July 2008: 5 were positive for *Bd* in both months; 10 were positive in April but negative in July; 2 were negative in both months. For the entire sample, the decrease in prevalence between April and July was significant (*z*_(1, 135)_ = 4.301; *P* = 1.70 × 10^−5^). Mean intensity of infection also declined significantly from GE 1295.9 (±924.3 SE, *n* = 46 infected hosts) to 38.9 (±11.4 SE, *n* = 42) (*t*_(1,81)_ = 4.039; *P* = 1.21 × 10^−4^).

For the total of 152 *X*. *laevis* tested in April and July 2008, sex and age were determined for 90%: ages ranged from nearly 2 years (33 individuals metamorphosed in summer 2006) to nearly 15 years (15 metamorphosed in summer 1993). Older adults (metamorphosed 1993–2002) and juveniles (metamorphosed in 2004 and 2005) occurred in all three combinations of infection status in the April and July recaptures. There was no significant effect of sex or age on either infection prevalence (Sex *z*_(1, 105)_ = 0.675; *P* = 0.499, Age *z*_(1, 121)_ = 1.493; *P* = 0.135), or mean intensity (Sex *t*_(1,69)_ = 1.212; *P* = 0.229, Age *t*_(1,61)_ = 0.757; *P* = 0.452), nor an effect of their interactions with sample date.

At the time of the field collections, water temperature was 8.5 °C in mid-April and 15 °C in early July. The temperature logger record for 2007/2008 showed that April 2008 ended a period of over 6 months when water temperature had been more-or-less continuously <10 °C and frequently <6 °C (minimum 2 °C) in mid-winter. The sample of *X*. *laevis* collected in July had experienced water temperatures ⩾15 °C for most of the preceding 3 months (see Tinsley et al., 2011a).

#### 3.2.2 Native amphibians

**Fig. 4:**
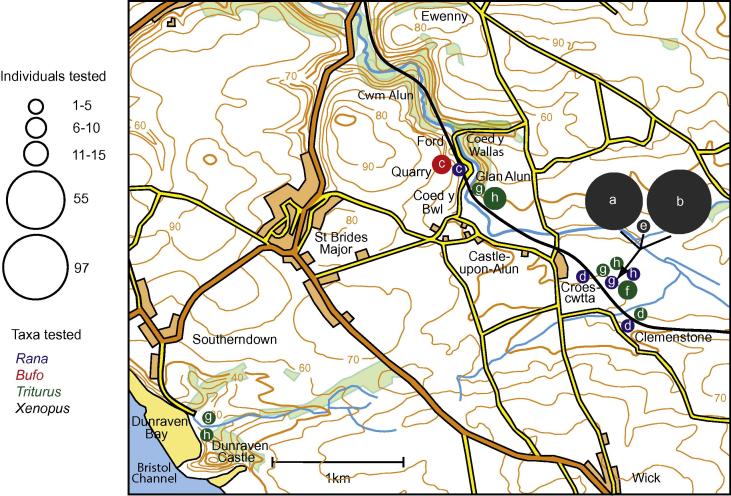
Location of habitats of amphibians tested with RT-PCR for infection by *Batrachochytrium dendrobatidis* in Wales; area centred on 51°27′50″N, 3°34′14″W, south of Bridgend, Glamorgan. Fieldwork periods each typically of 2–4 days in April (a), July (b), August (c) and September (d) 2008; May (e) and June (f) 2010; July (g) and September (h) 2011. Samples of species distinguished by symbol colour with numbers of individuals indicated by symbol size (see key) for native amphibians: frogs (*Rana temporaria*) in blue, toads (*Bufo bufo*) red, newts (‘*Triturus*’ comprising *Lissotriton vulgaris*, *Triturus helveticus* and *T*. *cristatus*) green; and for introduced *Xenopus laevis* black in the pond at Croescwtta Farm only.

Samples of native species in Wales tested for *Bd* by RT-PCR comprised 15 individuals in August and September 2008 (pre-extinction of the population of *X*. *laevis*) and 34 individuals in June 2010, July and September 2011 (post-extinction). Overall totals were 36 newts (including 2 great crested newts), 7 frogs (*Rana temporaria*) and 6 toads (*Bufo bufo*). Localities occur in three groups (Fig. 4): Croescwtta pond and adjacent habitats, 14 newts and 5 frogs; the quarry at Coed y Bwl and pond at Glan Alun cottage, 18 newts, 2 frogs and 6 toads; the marsh at Dunraven, 4 newts. The last 3 fieldwork sessions (June 2010, July and September 2011) included frogs (2), newts (12) and a great crested newt caught in the pond at Croescwtta recently vacated by the *Bd*-infected *X*. *laevis* (Section 3.2.1.). The Glan Alun pond supported a population of *X*. *laevis* from the start of records (early 1980s) continuing until at least the mid-2000s (Tinsley et al., submitted for publication). The present survey here (post-extinction) recorded a large breeding population of newts: for instance, 25 adults were caught in a single trap over 1 night in July 2011.

All except one of these native amphibians was negative for *Bd*. The single specimen infected (infection intensity: GE 23) was a juvenile *B*. *bufo* found at the Coed y Bwl quarry in August 2008. This occurred with 5 other toads and 2 frogs (*Bd* negative), found alongside 12 slow worms (*Anguis fragilis*) and a common lizard (*Lacerta vivipara*), all sheltering beneath pieces of fabric (floor covering or roof felt) distributed across an area of regenerating woodland. The coverings carried identification numbers/letters and may have been placed as refuges for a herpetological survey. One toad and 1 frog in this sample were adult (snout-vent-length (SVL) 5 and 6 cm respectively), the others were juveniles (SVL 1.5–3 cm): the smallest probably young-of-the-year, the largest probably aged 1 + years. The individual that was *Bd* positive had SVL 1.5 cm, consistent with metamorphosis in the weeks preceding capture.

### 3.3 Field studies in Lincolnshire

#### 3.3.1 Occurrence of infection in *Xenopus laevis*

**Fig. 5:**
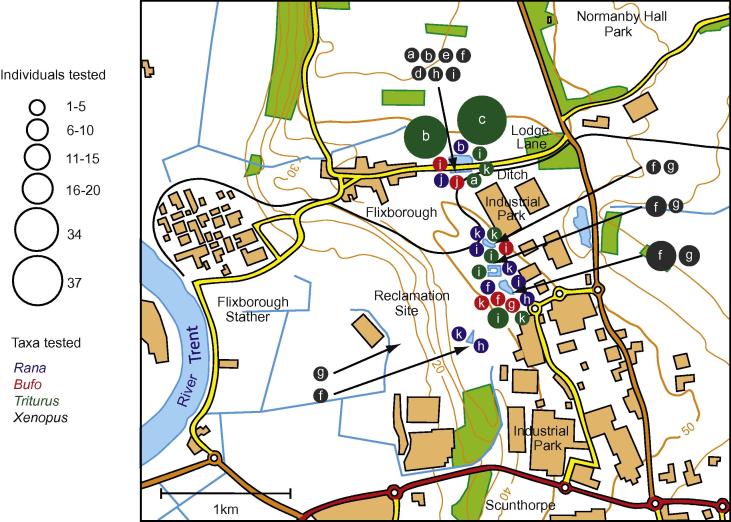
Location of habitats of amphibians tested with RT-PCR for infection by *Batrachochytrium dendrobatidis* in England; area centred on 53°37′30″N, 0°40′14″W, north of Scunthorpe, North Lincolnshire. Fieldwork periods each typically of 3–5 days in May (a), June (b), early July (c) and late July (d) 2008; June (e), July (f), August (g) and September (h) 2009; June (i), July (j) and August (k) 2010. Samples of species distinguished by symbol colour with numbers of individuals indicated by symbol size (see key) for native amphibians: frogs (*Rana temporaria*) in blue, toads (*Bufo bufo*) red, newts (‘*Triturus*’ comprising *Lissotriton vulgaris* and *T*. *helveticus*) green; and for introduced *Xenopus laevis* black with arrows to specific sites.

Tests with RT-PCR on captures in the ditch at Lodge Lane (Fig. 5) in May, June and July 2008 (*n* = 11), June, July and September 2009 (*n* = 11), and the last survivor caught in June 2010, were all negative for *Bd*. Samples from the reclamation site in 2009 included 32 *X*. *laevis* caught in late July and 13 in early/mid August: prevalence was 22% in July and zero 2 weeks later. Six of the 7 infected individuals had the lowest category of intensity, GE 0.1–1.0, and 1 had an infection of GE 10. Infection was present in almost all individual ponds shown in Fig. 5 and in adult female *X*. *laevis* (23.5%, *n* = 17), adult males (14.3%, *n* = 7) and juveniles (40%, *n* = 7) (sex unrecorded for 1), but the small subsamples precluded detailed comparisons. The sample uninfected in August comprised 7 adult females, 4 adult males and 2 juveniles caught in almost all the same ponds as the infected July sample.

#### 3.3.2 Native amphibians

Samples in Lincolnshire tested for *Bd* by RT-PCR included 85 individuals in May, June and July 2008, and July, August and September 2009 (pre-extinction of *X*. *laevis*) and 40 individuals in June and August 2010 (immediately post-extinction). Species totals comprised 100 newts, 14 frogs and 11 toads. Individuals were swabbed immediately after capture in two contiguous areas, corresponding with the focus of trapping of *X*. *laevis* (Fig. 5). At the Lodge Lane ditch, tests comprised 81 newts, 4 frogs and 4 toads. On the reclamation site, the totals were 19, 10 and 7 for these native taxa respectively, including frogs and toads caught at the same time and in the same habitats as the *X*. *laevis* found infected with *Bd*. All individuals of the native species were negative for *Bd*.

Night-time fieldwork showed that native amphibians were abundant across the study area especially in wetland habitats bordering the derelict industrial land. Abundance was illustrated by the captures of adult newts in traps set for *X*. *laevis* along ca. 50 m of the Lodge Lane ditch in 2008 (Fig. 5): 34 were caught during 3 nights of fieldwork in June and then 37 with similar trap effort in July. Water temperatures in the Lodge Lane ditch (Fig. 5) were closely comparable to those recorded in Wales (3.2.1); typically <10 °C for over 6 months/year (late October to early May) and ⩾15 °C for only 3–4 months (May/June to mid-September).

## 4 Discussion

This study presents the first evidence of *Bd* infection in introduced *X*. *laevis* populations in the U.K. It documents infections which probably persisted, at least in Wales, for over 50 years. A survey of U.K. laboratories showed low level *Bd* infection in most *X*. *laevis* colonies maintained for research. Experiments confirmed that infection levels in both laboratory and field conditions are strongly affected by environmental factors, particularly temperature. Although *X*. *laevis* is infected with Bd in several areas of global introduction, this is the first study to investigate the possibility of transfer of infection to sympatric native species. Fieldwork demonstrated the potential for transmission of *Bd* from *X*. *laevis* to co-occurring amphibians, but there was no evidence of infection associated with *X*. *laevis*, nor self-sustaining in native populations.

### 4.1 Characteristics of Bd infection in *Xenopus laevis*

Exports of *X*. *laevis* from South Africa for medical use began in the 1930s and studies on museum collections have shown that *Bd* was present in *X*. *laevis* at this time in the Western Cape, the source of most exports (Weldon et al., 2004). For many decades up to the present, wild-caught *X*. *laevis* are therefore likely to have arrived at laboratories worldwide carrying infection. Present studies show that these infections persist during long-term maintenance of laboratory populations. This has relevance for concerns that release or accidental escape of *X*. *laevis* might introduce *Bd* into the environment (Fisher and Garner, 2007). Survey results showing *Bd* in nearly 90% of laboratories suggest that pathogen introduction by this route, and possibly by waste water drainage, would be likely. Our findings also highlight the risk of cross-infection to other amphibian species co-housed in the same laboratory with *X*. *laevis*. Indeed, such a spill-over event has already been recorded for transfer of *Bd* from *Xenopus gilli* to the Mallorcan Midwife Toad, *Alytes muletensis*, maintained in the same captive-breeding facility (Walker et al., 2008).

While the origins of the *X*. *laevis* established in the U.K. are unknown, the founding members of the population in Wales (and also a population introduced in the early 1960s on the Isle of Wight but now probably extinct (Tinsley et al., submitted for publication)) were almost certainly wild-caught in South Africa. This conclusion is based on records of African helminth parasites in U.K. *X*. *laevis*. The monogenean *Protopolystoma xenopodis* is strictly specific to *X*. *laevis* (see Tinsley and Jackson, 1998); its presence in the Welsh population indicates that the parasite was introduced with infected hosts and then survived in Wales for about 50 years (Tinsley et al., 2012). The cestode *Cephalochlamys namaquensis* occurred in the population of *X*. *laevis* on the Isle of Wight until the 1980s (Jackson and Tinsley, 2001); this tapeworm therefore cycled for several decades while its host population existed. *Bd* infection in *X*. *laevis* in both Wales and Lincolnshire is likely to follow this pattern, suggesting that the pathogen was probably introduced either with hosts originally wild-caught in South Africa or with *X*. *laevis* raised in captivity but infected, ultimately, from imported progenitors; *Bd* then persisted continuously under U.K. climate conditions in successive generations of hosts until their extinction in 2010.

The survey of U.K. laboratories maintaining *X*. *laevis* showed that, typically, *Bd* was detectable in few individual animals in a colony alongside a majority that was *Bd* negative. The low prevalence (7%) emphasises the high level of immunity characteristic of *X*. *laevis* as a host of *Bd* (Rollins-Smith et al., 2009). In our experiment monitoring the same individuals over time (Section 3.1), prevalence was typically around 3% at each interval but different individuals were responsible at different times: these findings suggest that the low measures of prevalence recorded in laboratory and field studies may actually relate to a fluctuating subset of the population. However, overt infection is strongly determined by environmental conditions: factors associated with stress (including sudden temperature fluctuations and parasite infection) may precipitate increased prevalence and intensity. The major influence of low temperature is consistent with the general phenomenon of cold-induced immunosuppression in amphibians (Rollins-Smith et al., 2011). The temperature-dependency of *Bd* infection has been demonstrated for *Xenopus (Silurana) tropicalis* by Ribas et al. (2009): increased infection levels at suboptimal host temperatures may be attributable to impaired antimicrobial peptide activity and compromised immune defence. The present laboratory studies quantify the outcome of these temperature effects in *X*. *laevis*. Lab-raised offspring of a single family showed 100% prevalence at 10 °C but only 3% at 20 °C. This mirrored the outcome of transfer of wild-caught *X*. *laevis* from fluctuating cool temperatures in Wales (Tinsley et al., 2011a) to a laboratory environment with constant higher temperature: after >6 months at 20 °C, only 5% of individuals were Bd positive.

Fieldwork confirms that seasonal temperature changes are associated with equivalent effects in the wild: prevalence in the Welsh population was twice as high in April (84%) as in July (43%), with major differences in individual intensities. The two samples differed in water temperature at the time of collection: 8.5 °C in April and 15 °C in July. However, the environmental conditions affecting infection status may be better defined by the thermal regime in the period preceding sampling. The animals heavily-infected in April 2008 had experienced temperatures <10 °C for over 6 months, while the reduced infections in July occurred after 3 months at ⩾15 °C. Although other environmental changes will also have occurred between these sampling dates, a major effect of temperature is supported by the parallel laboratory results. Similar very high intensities (including 100,000 GE) occurred at 8.5 °C in the field (Fig. 3a) and 10 °C in the laboratory (Fig. 2a).

This study detected no significant effect of age or sex on field infections. However, the power to detect an age effect on infection intensity or infection probability, which might have been indicative of acquired immunity, was probably relatively low because there were few mid-aged individuals in the samples. The fieldwork added a further dimension to documenting the behaviour of *Bd* in the wild based on repeat recaptures of the same individually-marked animals. Of the 15 individuals positive for *Bd* in April, 10 had lost their infection by July. No individuals that were free of *Bd* in April gained infection with the temperature increase.

Other studies on *X*. *laevis* have recorded low prevalence in warm climate environments, in South Africa and California: typically 2–4% (Vredenburg et al., 2013; Weldon et al., 2004). But the corollary of this is that a few infected individuals are evident in each data set (e.g. 1 in 35 and 1 in 21 at 20 °C in present studies, Figs. 2b and 3c). Repeat testing of lab colonies also revealed a minority of individuals with persistent infections. These could represent reservoirs of infection with less effective immunity, responsible for transmitting to others within a population during conditions promoting re-activation. However, it is unclear whether *X*. *laevis* recorded as *Bd*-negative by RT-PCR are entirely chytrid-free or whether they carry undetectable infections. This has relevance for the observed fluctuations in infection status: when *Bd*-negative individuals convert to *Bd*-positive (especially in response to stress), it is unresolved whether these have acquired new infection from other hosts or whether an existing hidden infection has re-emerged in the individual following immune suppression or other environmental effects.

In Lincolnshire, the lack of recorded infection in Lodge Lane ditch could reflect small sample size but the eradication programme had reduced *X*. *laevis* numbers at this site from over 600 adults and juveniles in 2003 to only 11 in both 2008 and 2009 (Tinsley et al., submitted for publication): *Bd* infection could have been lost by chance following removal of those individuals most susceptible. On the reclamation site, almost all individuals in the infected component (22%) of the July 2009 sample had the lowest detectable levels of *Bd*. The absence of recorded infection in the following month could be an artefact of small sample size (*n* = 13), but it might indicate development of immunity in these individuals accompanying increased summer temperatures.

Swabs were taken with standard precautions to prevent contamination during fieldwork, but individuals were in contact with one another naturally in the pond before capture and then closely confined inside traps before swabbing. However, lab studies confirmed that transfer of detectable levels of *Bd* between *X*. *laevis* does not occur in these circumstances: thus, our monitoring of individuals housed in groups of 4 showed that, typically, infection remained restricted to single individuals during several months of habitat sharing (Section 3.1 and Supplementary Table 2). Since *Bd* was not recorded on native species collected in water, the possibility of chance contamination from co-occurring *X*. *laevis* did not arise.

### 4.2 The potential for transfer of infection to native amphibian populations

The field and laboratory findings that low temperatures may precipitate high prevalence and intensity of *Bd* indicate that *X*. *laevis* populations in cool temperate regions are likely to carry heavy infections for considerable periods seasonally (potentially ⩾ 6 months/year based on water temperatures <10 °C in Wales). The threat to native amphibians is further increased because habitat overlap with *X*. *laevis* is maximal during spring when native species may congregate to spawn in ponds where *Bd* infections in *X*. *laevis* would be heaviest. This elevated risk will have been repeated every year since the original introductions, probably for over 20 years in Lincolnshire and 50 years in Wales (Tinsley et al., submitted for publication). Thus, the present ‘case study’ of *Bd* infection in U.K. *X*. *laevis* represents an exacting test of the potential for establishment of infection in native species.

Investigation of native species involved an overall total of 174 individuals and interpretation is further strengthened by the spatial and temporal scope of the survey. In both Wales and Lincolnshire, native amphibians were sampled throughout the recorded ranges of the respective populations of *X*. *laevis*, giving complete cover of recently-known habitats, testing for on-going transmission from co-occurring *X*. *laevis*. In Wales, the geographical coverage extended additionally across the area occupied by *X*. *laevis* over the 30 years of the mark-recapture programme. At some of these localities, *X*. *laevis* had not been seen since the 1980s (e.g. Coed y Bwl quarry), the mid-1990s (Dunraven) or mid-2000s (Castle-upon-Alun) (Tinsley et al., submitted for publication). So, the survey here tested for a legacy of *Bd* from previous periods of co-existence, persisting after the disappearance of the original source. In both areas, sampling covered the years pre- and post-dating the extinction of *X*. *laevis* and included individuals caught alongside *X*. *laevis* shown to carry *Bd*.

In Lincolnshire, the survey of *R*. *temporaria*, *B*. *bufo* and *T*. *vulgaris* and/or *T*. *helveticus* found no *Bd* infection in the study area. This conclusion was based on tests of 125 animals sampled during 3 years in the habitats occupied by *X*. *laevis*. In Wales, a parallel survey over 4 years sampled the same native species together with *T*. *cristatus*, totalling 49 individuals. A single positive case, in *B*. *bufo*, was recorded; this was confirmed as a true infection by the GE estimate of 23. However, the record has some unusual features. The site is 2 km from the only locality (Croescwtta) with *Bd* recorded in *X*. *laevis* at that time (2008). *X*. *laevis* did occur at the Coed y Bwl quarry but disappeared in the late 1980s when a pond in which *X*. *laevis* and native amphibians spawned was filled in (Tinsley et al., submitted for publication). In 2008, there were no obvious sites in the vicinity where this recently-metamorphosed toad could have developed as a tadpole and it seems unlikely that such a small individual (SVL 1.5 cm) could have migrated here from a distant spawning site in the few weeks since metamorphosis. There were also no known sources from which this individual could have acquired infection: frogs, toads and newts sampled in this part of the Alun valley (*n* = 26) were otherwise all negative (Fig. 4). The discovery of the quarry sample beneath numbered sheets of fabric suggested that refuges had been constructed for ecological monitoring. Without a convincing local origin for this single *Bd* record, it is possible that the infection resulted from contamination with the imported refuge material, or from visits by workers involved with herpetological studies elsewhere, or by other vectors/reservoirs including wildfowl (e.g. Garmyn et al., 2012). However, local naturalists reported that the Coed y Bwl quarry has been used as a site for translocation of amphibians and reptiles rescued from construction development projects elsewhere in Glamorgan. So, the assemblage of frogs and toads (in at least 3 age classes: post-metamorphs, juveniles and adults), together with the lizards, may have arrived at this site as part of a group. It seems most probable that the isolated case was introduced from outside the Alun valley and was entirely independent of *X*. *laevis*.

The absence of other *Bd* cases suggests that there are no self-sustaining *Bd* populations in native amphibians in the two study areas. But, it is possible for on-going transmission to be masked by overwhelming mortality: a highly virulent pathogen could cause rapid host death and leave little record in prevalence measurements. However, the continuing abundance of each of the native amphibians in Wales and Lincolnshire was illustrated by their frequent captures in traps set for *X*. *laevis* (Sections 3.2.2, 3.3.2 and Figs. 4 and 5). Large population numbers were particularly striking for newts that might be at greatest risk, co-occurring in water with *X*. *laevis* for the longest periods each year. Similar indications of abundance were recorded both before and after the extinction of the respective *X*. *laevis* populations, especially on the industrial wasteland in Scunthorpe. So, the evidence of this study suggests that there have been no detectable negative effects on native species at a population level.

### 4.3 Conclusions

The most conclusive indication of failure of *Bd* to establish in native populations comes from habitats where local amphibians co-occurred with *X*. *laevis* known to carry chytrid infection. Sites in both Wales (Croescwtta) and Lincolnshire (the reclamation site) provide this test. In our overall sample of 174 native amphibians, no *Bd* was recorded in the subset of 55 individuals from those localities where co-occurrence was recorded and exposure to cross-infection over many years would have been most intense. These data from our 4 year study must still be interpreted cautiously, constrained by sample size, the sensitivity of swab testing, and the finding that individuals of some susceptible species may carry fluctuating low-level infection (Briggs et al., 2010). However, concerns that introduced *X*. *laevis* might have long-term population consequences for native amphibians are not substantiated for the two study areas. If significant negative effects had occurred then these should have been evident after exposure to *Bd* during several decades (up to half a century for the population in Wales). Solis et al. (2010) recorded *Bd* in introduced *X*. *laevis* in Chile and, whilst emphasising the need for greater monitoring, observed that no declines of native anurans had been noted in areas of co-occurrence after almost 40 years. Nevertheless, whatever the putative risk for native amphibians created by *Bd*-infected *X*. *laevis*, that risk has now ended in the U.K. with the extinction of these introduced populations. The apparently neutral outcome could be specific to the environmental conditions and the native species ‘at risk’ in the two areas of the U.K. Some anuran species can co-exist with *Bd* and, within a species, some populations co-exist with *Bd* when others decline to extinction (Briggs et al., 2010). This heterogeneity in *Bd* epidemiology may be influenced by genotypic virulence variation of *Bd*, host immunological responses, and by complex interactions between host and pathogen phenotypes, the environment, antimicrobial peptides, skin bacteria and aquatic microfauna (Fisher et al., 2009; Gervasi et al., 2013, 2014; Lam et al., 2010; Rollins-Smith et al., 2011; Schmeller et al., 2014). Laboratory experiments also suggest the possibility of acquired behavioural and immunological resistance to *Bd* in some amphibians (McMahon et al., 2014). It is possible that the *Bd* lineage introduced with *X*. *laevis* into the U.K. is poorly-adapted to native amphibians and environmental conditions: genotyping the lineages circulating in *X*. *laevis* populations would provide valuable epidemiological information on virulence. The introduced populations of *X*. *laevis* in other geographical regions, with other combinations of native amphibians and chytrid isolates, represent separate ‘natural experiments’ in the transfer of *Bd* infection: the role of *X*. *laevis* in these environments requires case-by-case investigation.
