# Supplementary material for: Chytrid fungus infections in laboratory and introduced Xenopus laevis populations: assessing the risks for U.K. native amphibians
Source: Biol Conserv. 2015 Apr;184:380–8. doi: 10.1016/j.biocon.2015.01.034 (PMC4380136; doi:10.1016/j.biocon.2015.01.034)
Supplement: Supplementary data 1 [file mmc1.docx]

**A.**

**Supplementary information: Methods**

Fieldwork procedures

*Xenopus laevis* were caught with baited traps in the range of habitat-types occupied in Wales and Lincolnshire: ponds, marshes and ditches with static water and (in Wales only) streams and rivers with flowing water. The traps, equipped with a funnel entrance, were of 2 designs: collapsible tapered cylinders of nylon netting (5 mm mesh) typically 1–3 m long and 35 cm diameter at the wide end, supported on a hooped frame; plastic buckets 43 cm long, 30 cm diameter, with lids in which an entrance hole had been cut. The funnels for the net traps were tapered netting tubes 30–40 cm long; funnels for the buckets were stainless steel cones 16 cm long set into the bucket lid; both had an internal opening of about 6 cm diameter. Netting traps could be used in deep water, completely submerged along most of their length, with the narrow end extending out of water so that trapped animals had access to air. Buckets were set on their sides, partly submerged, with the entrance to the funnel below the water surface and air holes above the surface.

Raw liver and/or steak was used as bait, enclosed in muslin bags to prevent ingestion (ingestion would have distorted body weight measurements after capture and might have reduced subsequent attraction to bait if animals had fed and then escaped).

Traps were set from the onset of darkness (in summer from around 22.00h, earlier in spring/ autumn) and examined at dawn (beginning at around 04.00h in summer, later in spring/autumn). For some studies in Wales primarily concerned with helminth parasite infection (e.g. Tinsley at al., 2012), traps were typically set for 5h (from 21.00 – 02.00h) on a single fieldwork night. In other field studies, in Wales and Lincolnshire, after inspection at dawn, traps were re-set for the daytime period and then inspected again and re-set with fresh bait at dusk. These repeat trappings were continued for up to 6 successive sessions at specific sites; alternatively, the traps were moved to new locations each day during fieldwork periods of up to 5 days. Traps were set at relatively high density, typically 1.5–2 m apart along the banks of rivers and ponds. Some *X. laevis* were captured with long-handled pond nets in open water at night.

Animals caught were sexed, measured (snout-vent length) and weighed. In Wales, all individuals were marked at first capture with a unique identifier. In initial studies (1980s, early 1990s), marks were combinations of dye spots introduced on the light skin of the ventral surface with a dye injector (Panjet: Wright Health Group Ltd., Dundee). Alcian blue was found to be effective and long-lasting. In later studies, copper or stainless steel wire, cooled in liquid nitrogen, was applied to the ventral skin for 2–3 sec to create a freeze brand (Daugherty, 1976). In the final 10 years of fieldwork, these marks were 3 digit combinations of letters and numbers. Digital photographs of each mark were recorded to confirm identity at future captures. In no case (out of over 2000 markings for field and lab studies) was any injury observed and the repeat recaptures of marked individuals over subsequent years confirmed that this and associated fieldwork procedures had no detectable effect on survivorship. Thus, Tinsley et al. (2012) reported trapping in Wales on 20 occasions over a 10 year period during which the same marked individuals were caught on up to 19 of these occasions. These and other data indicate the potential for long survival (at least in Wales) but they also demonstrate that the marks remained legible for many years (up to a maximum of 15 years for the freeze brands and 23 years for the alcian blue dye marks).

For age determination, the distal 2 phalanges (approx. 2–3 mm) of one hind limb digit (usually the longest) were removed and fixed in formol saline or Bouin’s solution. The fixed bone was processed with standard histological procedures, including decalcification and embedding in paraffin wax, with microtome sections 5–8 µm thick attached to glass slides, de-waxed, stained with haematoxylin and eosin, and mounted as permanent preparations in Canada balsam. In both Wales and Lincolnshire, counts of annual growth rings were not affected by bone resorption.

Animals were tested for *Batrachochytrium dendrobatidis* with sterile swabs following procedures detailed in Hyatt et al. (2007): swabs were stroked repeatedly over the venter with special emphasis on the footwebs, axillary and inguinal regions. Single-use disposable gloves were used for handling each animal. Swabs were sealed in tubes with air-tight closures and stored at room temperature (typically 20°C) until assayed with RT-PCR. All fieldwork equipment (including nets, traps, containers, footwear) was sterilised between visits to different localities and repeat visits to the same sites, ensuring complete drying of all surfaces (usually with exposure to sunshine).

Samples of the native amphibian populations at the fieldwork sites (*Rana temporaria, Bufo bufo,* and *Triturus and Lissotriton* species) were swabbed following the same procedures. For each of these species, individuals were most often found within the traps baited for capture of *X. laevis.* Additionally, native amphibians were caught with pond nets in water and by hand on land adjacent to the water bodies surveyed at night for *X. laevis.* Some samples were collected during searches of suitable habitats and refuges by day.

Field and associated laboratory procedures were approved by the Ethics Committee at Bristol University and carried out under UK Home Office Licence (with the designated field sites in Wales registered by the Home Office).

**References**

Daugherty, C.H. (1976) Freeze-branding as a technique for marking anurans. *Copeia*, **1976**, 836-838.

Hyatt, A.D., Boyle, D.G., Olsen, V., Boyle, D.B., Berger, L., Obendorf, D. *et al.* (2007) Diagnostic assays and sampling protocols for the detection of *Batrachochytrium dendrobatidis.*  *Diseases of Aquatic Organisms*, **73**, 175-192.

Tinsley, R.C., Stott, L.C., York, J.E., Everard, A.L.E., Chapple, S.J., Jackson, J.A. *et al*. (2012) Acquired immunity protects against helminth infection in a natural host population: long-term field and laboratory evidence. *International Journal for Parasitology*, **42**, 931-938.
