# Supplementary material for: Chytrid fungus infections in laboratory and introduced Xenopus laevis populations: assessing the risks for U.K. native amphibians
Source: Biol Conserv. 2015 Apr;184:380–8. doi: 10.1016/j.biocon.2015.01.034 (PMC4380136; doi:10.1016/j.biocon.2015.01.034)
Supplement: Supplementary data 2 [file mmc2.docx]

**B.**

**Supplementary information: Table 1**

Twenty-two of the 27 laboratories that provided swabs from their captive *Xenopus laevis* for *Bd* testing also sent details about how their colonies were kept (tabulated below). Samples from these laboratories showed small numbers of individual frogs that had between 5 and 100 genome equivalents of *Bd* DNA as detected using the standard Taqman assay. With little variation in prevalence (overall 7.2% of individuals *Bd* positive), there was no relationship between the numbers of infected frogs or levels of infection and any of the care parameters recorded. There were 2 exceptions with high infection levels that were associated with recent unusual stress (see text).

| **Institute** | **System** | **Water** | **Food** | **Density (L/frog)** | **Total population** |
| --- | --- | --- | --- | --- | --- |
| A | Fill and Dump | Stood | Meat - Pellets | 9 | 400 |
| B | Recirculating | R/O | Pellets | 6 | 1000 |
| C | Recirculating | R/O | Pellets | 4 | 40 |
| D | Recirculating | Filtered | Meat | 2.5 | 50 |
| E | Recirculating | R/O | Pellets | 3 | 132 |
| F | Fill and Dump | Stood | Pellets | 8 | 40 |
| G | Fill and Dump | Tap | Meat - Pellets | 12 | 61 |
| H | Recirculating | Filtered | Pellets | 20 | 80 |
| I | Recirculating | Filtered | Pellets | 5 | 550 |
| J | Fill and Dump | Filtered | Frog Brittle | 3.8 | 160 |
| K | Fill and Dump | Filtered | Pellets | 2.5 | 4 |
| L | Fill and Dump | Filtered | Pellets | 7 | 20 |
| M | Recirculating | Filtered | Pellets | 6 | 24 |
| N | Recirculating | Filtered | Pellets | 3 | 600 |
| O | Fill and Dump | Filtered | Pellets | 10 | 400 |
| P | Fill and Dump | Stood | Meat | 5 | 150 |
| Q | Fill and Dump | Filtered | Pellets | 2.5 | 485 |
| R | Fill and Dump | Filtered | Pellets | 5 | 265 |
| S | Recirculating | R/O | Pellets | 2.5 | 1800 |
| T | Fill and Dump | Stood | Pellets | 2.5 | 140 |
| U | Fill and Dump | Stood | Pellets | 5 | 150 |
| V | Fill and Dump | R/O | Pellets | 4 | 500 |
