# Supplementary material for: Chytrid fungus infections in laboratory and introduced Xenopus laevis populations: assessing the risks for U.K. native amphibians
Source: Biol Conserv. 2015 Apr;184:380–8. doi: 10.1016/j.biocon.2015.01.034 (PMC4380136; doi:10.1016/j.biocon.2015.01.034)
Supplement: Supplementary data 3 [file mmc3.docx]

**C.**

**Supplementary information: Table 2**

Adult *Xenopus laevis* from the main European *Xenopus* Research Centre stock, which has a mixed background of NASCO animals and the offspring of frogs caught in the wild in the 1950s, were maintained at 18.5°C in 27 litre tanks in a recirculating water system (Tecniplast Ltd) as groups of 4 animals of a single sex. They were fed high protein pellets 3-5 times daily. Individual animals were identified using photographs of the distinctive patterns on their backs and were swabbed (see Methods) at the start of the periods shown. Taqman assays were used to identify animals with *Bd* (see text): positive tests shown with +. A positive control sample was included with each set of assays.

|  | Week |  | 1 | 3 | 5 | 6 | 7 | 8 | 10 | 11 | 12 | 13 | 19 |
| --- | --- | --- | --- | --- | --- | --- | --- | --- | --- | --- | --- | --- | --- |
| Tanks | Female | 1 |  |  |  |  |  |  |  |  |  |  |  |
|  |  | 2 |  |  |  | + |  |  |  |  |  |  |  |
|  |  | 3 |  |  |  |  |  |  |  |  |  |  |  |
|  |  | 4 |  |  |  |  |  |  |  |  |  |  |  |
|  | Female | 5 |  |  |  |  |  |  |  |  |  |  |  |
|  |  | 6 |  |  |  |  |  |  |  |  |  |  |  |
|  |  | 7 |  |  |  |  |  |  |  |  |  |  | + |
|  |  | 8 |  |  |  |  |  |  |  |  |  |  |  |
|  | Female | 9 |  |  |  |  |  |  |  |  |  |  |  |
|  |  | 10 |  |  |  |  |  |  |  |  |  |  |  |
|  |  | 11 |  |  |  |  |  |  |  |  |  |  |  |
|  |  | 12 |  |  |  |  |  |  |  |  |  |  |  |
|  | Female | 13 |  |  |  |  |  |  |  |  |  |  | + |
|  |  | 14 |  |  |  |  |  |  |  |  |  |  |  |
|  |  | 15 |  |  |  |  |  |  |  |  |  |  |  |
|  |  | 16 |  | + |  |  |  |  |  |  | + | + | + |
|  | Male | 17 |  |  |  |  |  |  |  |  |  |  |  |
|  |  | 18 |  |  |  |  |  |  |  |  |  |  |  |
|  |  | 19 |  |  |  |  |  |  |  |  |  |  |  |
|  |  | 20 |  |  |  |  |  |  |  |  |  |  |  |
|  | Male | 21 |  |  |  |  |  |  |  |  |  |  |  |
|  |  | 22 |  |  |  |  |  |  |  |  |  |  |  |
|  |  | 23 | + |  |  |  |  |  |  | + |  |  |  |
|  |  | 24 |  |  |  |  |  |  |  |  |  |  |  |
|  | Male | 25 |  |  |  |  |  |  |  |  |  |  |  |
|  |  | 26 |  |  |  |  |  |  |  |  |  |  |  |
|  |  | 27 |  |  |  |  |  |  |  |  |  |  |  |
|  |  | 28 |  |  |  |  |  |  |  |  |  |  |  |
|  | Male | 29 |  |  |  |  |  |  |  |  |  |  |  |
|  |  | 30 |  |  |  |  |  |  |  |  |  |  |  |
|  |  | 31 |  |  |  |  |  |  |  |  |  |  |  |
|  |  | 32 |  |  |  |  |  |  |  |  |  |  |  |
